# An optimized machine learning framework for predicting and interpreting corporate ESG greenwashing behavior

**DOI:** 10.1371/journal.pone.0316287

**Published:** 2025-03-06

**Authors:** Fanlong Zeng, Jintao Wang, Chaoyan Zeng

**Affiliations:** 1 School of Foreign Studies, Yiwu Industrial and Commercial College, Jinhua, Zhejiang, China; 2 School of Finance, Shanxi Technology and Business University, Taiyuan, Shanxi, China; 3 Graduate School, Lyceum of the Philippines University-Batangas, Batangas, Philippines; Atlantic Technological University, IRELAND

## Abstract

The accurate prediction and interpretation of corporate Environmental, Social, and Governance (ESG) greenwashing behavior is crucial for enhancing information transparency and improving regulatory effectiveness. This paper addresses the limitations in hyperparameter optimization and interpretability of existing prediction models by introducing an optimized machine learning framework. The framework integrates an Improved Hunter-Prey Optimization (IHPO) algorithm, an eXtreme Gradient Boosting (XGBoost) model, and SHapley Additive exPlanations (SHAP) theory to predict and interpret corporate ESG greenwashing behavior. Initially, a comprehensive ESG greenwashing prediction dataset was developed through an extensive literature review and expert interviews. The IHPO algorithm was then employed to optimize the hyperparameters of the XGBoost model, forming an IHPO-XGBoost ensemble learning model for predicting corporate ESG greenwashing behavior. Finally, SHAP was used to interpret the model’s prediction outcomes. The results demonstrate that the IHPO-XGBoost model achieves outstanding performance in predicting corporate ESG greenwashing, with *R²*, *RMSE*, *MAE*, and *adjusted R²* values of 0.9790, 0.1376, 0.1000, and 0.9785, respectively. Compared to traditional HPO-XGBoost models and XGBoost models combined with other optimization algorithms, the IHPO-XGBoost model exhibits superior overall performance. The interpretability analysis using SHAP theory highlights the key features influencing the prediction outcomes, revealing the specific contributions of feature interactions and the impacts of individual sample features. The findings provide valuable insights for regulators and investors to more effectively identify and assess potential corporate ESG greenwashing behavior, thereby enhancing regulatory efficiency and investment decision-making.

## 1. Introduction

In recent years, ESG issues have increasingly become a focal point of global attention, with investors, regulatory bodies, and consumers placing greater emphasis on the sustainable development performance of enterprises. In March 2024, the People’s Bank of China issued the “Guiding Opinions on Further Strengthening Financial Support for Green and Low-Carbon Development,” which emphasized the need to strengthen constraint mechanisms based on information disclosure. This directive aimed to guide listed companies to disclose sustainable development information, thereby highlighting the importance of ESG information disclosure in China. However, some companies with poor actual ESG performance manipulate selective disclosure of ESG data to create a false image of sustainability, seeking market recognition and investor trust. Yu et al. [1] refer to this as corporate “greenwashing” behavior, which can lead to a series of greenwashing risks. Therefore, researching corporate ESG greenwashing behaviors is of significant importance for reducing related risks, enhancing information transparency, and improving regulatory effectiveness.

Regarding corporate ESG greenwashing behaviors, studies by Tashman et al. [2] and Hora et al. [3] have pointed out that there is often a discrepancy between ESG statements and actual performance in practice, indicating the prevalence of corporate ESG greenwashing. Liu et al. [4] identified external governance environments and internal governance mechanisms as the two main drivers of corporate ESG greenwashing behaviors. For example, the implementation of green credit policies can initially lead to a significant increase in greenwashing behaviors [5]; additionally, financially constrained companies or those with high debt levels may resort to greenwashing due to future investment and financing needs [6,7]. The drivers of corporate ESG greenwashing are varied, as are the risks. Greenwashing behaviors can mislead investors about the actual ESG performance of enterprises [8], resulting in erroneous investment decisions and potentially leading to incorrect stock market pricing [9]. Furthermore, it can undermine the market recognition and capital support deserved by genuinely sustainable enterprises, reducing market fairness and transparency [10]. To mitigate various greenwashing risks, a reliable greenwashing identification and evaluation mechanism is needed. Consequently, various methods for measuring greenwashing have emerged [1,11–15]. However, these studies primarily focus on ex-post identification of corporate greenwashing behaviors, with few addressing the prediction of corporate ESG greenwashing behaviors. Accurate prediction of corporate greenwashing behaviors, as opposed to ex-post identification, can better assist regulatory agencies in monitoring corporate ESG disclosure behaviors, enhancing market transparency, and protecting investor interests. Therefore, developing effective prediction models for corporate ESG greenwashing behaviors holds significant theoretical and practical importance.

Machine learning, with its powerful data processing and prediction capabilities, has become a popular tool for addressing prediction problems in various fields such as management decision-making [16,17], transportation [18], healthcare [19], agriculture [20], climate change [21,22], and construction engineering [23,24]. Classical machine learning algorithms include Linear Regression (LR), Support Vector Machines (SVM), Random Forest (RF), Artificial Neural Networks (ANN), and XGBoost. XGBoost, an ensemble learning algorithm based on Gradient Boosting Decision Trees (GBDT), was proposed by Chen et al. [25]. Due to its efficient computational performance and excellent predictive capabilities, XGBoost has gained widespread recognition in academia and industry. Niazkar et al. [26] systematically summarized the applications of XGBoost in the field of water resources engineering from December 2018 to May 2023; Meddage et al. [27–29] have extensively applied the XGBoost model to various predictive problems in the field of construction engineering; Shi et al. [30] investigated early prediction of acute kidney injury based on the XGBoost model; and Wang et al. [31] verified the superiority of the XGBoost model compared to K-Nearest Neighbors (KNN), SVM, RF, and Back Propagation Neural Network (BPNN) models in predicting e-commerce user purchasing behavior.

However, machine learning models generally suffer from poor interpretability, and XGBoost is no exception. To address this issue, several studies have explored the application of Explainable Artificial Intelligence (XAI) techniques. For example, Ukwaththa et al. [32] reviewed machine learning and XAI methods in additive manufacturing, highlighting the importance of interpretability in complex models. Similarly, Perera et al. [33] applied XAI techniques to streamflow modeling in ungauged basins, using local interpretable model-agnostic explanations to interpret a modified generative adversarial network model. These studies emphasize the growing use of XAI methods to improve the transparency and interpretability of machine learning models. The SHAP theory, proposed by Lundberg et al. [34], is one of the XAI techniques.SHAP, as a unified framework for enhancing the interpretability of machine learning models, enables both global and local model interpretability analyses, helping users understand the contribution of each feature to the prediction results [35]. Consequently, scholars from different research fields have begun combining XGBoost with SHAP. For example, Wu [36] used the XGBoost-SHAP framework to predict and explain the economic resilience of Chinese provinces, identifying R&D expenditure and patent authorizations as key factors influencing economic resilience in China. Yi et al. [37] employed this framework to predict the difficulty of mathematics exam questions in the field of education, finding that parameter-level and reasoning-level features were crucial factors influencing the difficulty of subjective exam questions. Notably, the XGBoost-SHAP framework is more commonly used in transportation prediction problems [38–40]. These applications demonstrate the maturity and broad applicability of the XGBoost-SHAP research framework.

Nevertheless, in practical applications, the predictive performance of the XGBoost model heavily depends on the choice of hyperparameters such as n_estimators, learning_rate, and max_depth. The interaction between these hyperparameters is highly complex, as their values not only affect the model’s learning ability but also its generalization performance. The optimization of this hyperparameter space is crucial for achieving the best predictive results. Previous studies have shown that the performance of tree-based models like XGBoost is sensitive to the settings of key hyperparameters, and optimizing these parameters can lead to significant improvements in accuracy and generalization ability [25,41–45]. Common hyperparameter optimization methods include Grid Search (GS) and Bayesian Optimization (BO). Grid Search exhaustively searches all possible parameter combinations to find the optimal solution, which, while simple and straightforward, is computationally expensive and inefficient [41]. Bayesian Optimization guides the search process by constructing a surrogate model, which is more efficient than Grid Search but may still encounter local optima issues in high-dimensional spaces [42]. Recently, some studies have attempted to use various intelligent optimization algorithms (such as Genetic Algorithms (GA) [43], Particle Swarm Optimization (PSO) [44], and Whale Optimization Algorithm (WOA) [45]) for XGBoost hyperparameter tuning, achieving certain successes. The Hunter-Prey Optimization (HPO) algorithm, proposed by Naruei et al. [46], is an emerging intelligent optimization algorithm with strong global search capabilities and convergence accuracy. However, the original HPO algorithm still has certain limitations when dealing with complex optimization problems [47]. To improve optimization efficiency and performance, this study proposes an IHPO algorithm, making it more suitable for XGBoost hyperparameter optimization. By incorporating Tent Chaos Mapping and Lévy distribution, the algorithm enhances the quality of the initial population and global search capabilities. Additionally, an adaptive inertia weight strategy is adopted to improve the balance during the optimization process and accelerate convergence.

**Fig 1:**
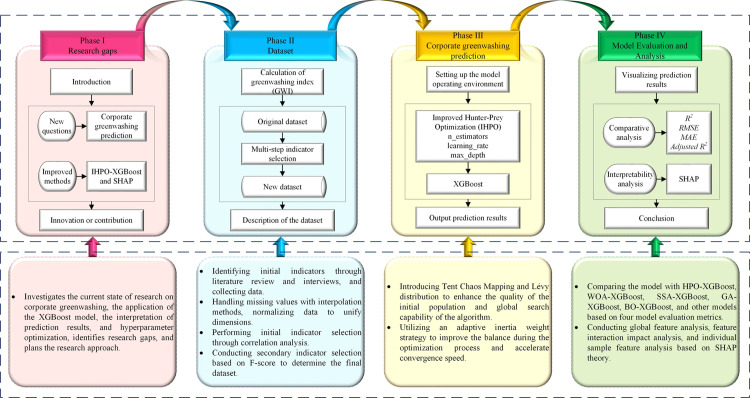
Research framework.

In summary, existing research lacks focus on the prediction of corporate ESG greenwashing behaviors, and there are still deficiencies in hyperparameter optimization and interpretability of the XGBoost model. To address these issues, this study undertakes the following tasks to pave the way for further research on corporate ESG greenwashing prediction. First, ESG-related data of Chinese A-share listed companies from 2017 to 2022 were collected, and an ESG greenwashing prediction indicator system was constructed through indicator screening, resulting in a corporate greenwashing prediction dataset. Then, the improved IHPO algorithm was applied to optimize the hyperparameters of the XGBoost model to enhance its predictive performance. Next, the optimized XGBoost model and the corporate greenwashing prediction dataset were used to predict corporate ESG greenwashing behaviors. Finally, the effectiveness of the proposed IHPO-XGBoost model was validated through model comparison analysis, and the prediction results were interpreted using SHAP theory. The specific research steps are illustrated in Fig 1. The potential contributions of this study are:

(1) Exploring the new issue of corporate ESG greenwashing prediction.
(2) Proposing an IHPO-XGBoost-based corporate ESG greenwashing prediction model that significantly enhances the predictive performance of the XGBoost model through hyperparameter optimization.
(3) Enhancing model interpretability and transparency by interpreting corporate greenwashing prediction results using SHAP theory, deepening stakeholders’ understanding of greenwashing behaviors, and providing tools for regulators and investors to identify and evaluate corporate ESG greenwashing behaviors.
(4) Offering a machine learning framework with certain generalizability and application value that can serve as a reference for studies in other fields.

## 2. Dataset

### 2.1. Measurement of corporate greenwashing index

To study the prediction of corporate ESG greenwashing behaviors, it is first necessary to determine how to quantify the degree of greenwashing by enterprises. The literature [1,13–15] provides a relatively consistent method for quantifying corporate greenwashing, which has been maturely applied in related research. Therefore, this paper refers to these studies and uses Equation (1) to calculate the greenwashing index (GWI).


$$GWI_{i,t}=\left(\frac{ESG_{dis\;i,t}-\bar{ESG_{dis}}}{\sigma_{dis}}\right)-\left(\frac{ESG_{per\;i,t}-\bar{ESG_{per}}}{\sigma_{per}}\right)$$ (1)

Where $ESG_{dis\;i,t}$ represents the ESG disclosure score of company *i* at time *t*, $\bar{ESG_{dis}}$ and $\sigma_{dis}$ are the mean and standard deviation of the ESG disclosure scores of companies in the same industry, respectively. $ESG_{per\;i,t}$ represents the actual ESG performance score of company *i* at time *t*, $\bar{ESG_{per}}$ and $\sigma_{per}$ are the mean and standard deviation of the actual ESG performance scores of companies in the same industry, respectively. If the value calculated according to Equation (1) is greater than 0, it indicates that the company is engaging in greenwashing, and the larger the value, the greater the degree of greenwashing.

In practical operation, this paper uses the Bloomberg ESG rating as the ESG disclosure score and the Wind ESG rating as the actual ESG performance score. The rationale for this choice is that the Bloomberg ESG rating encompasses a broad range of information disclosures on environmental, social, and governance aspects, serving as an important means for companies to demonstrate their ESG commitments externally. On the other hand, the Wind ESG rating is based on actual operational data of the company, reflecting the true performance of the company in terms of environmental protection, social responsibility, and governance structure.

### 2.2. Feature variables

This study employs multiple steps to determine the feature variable set for predicting corporate ESG greenwashing behaviors. The specific steps are as follows:

Step 1: Through extensive literature review [4–7,48–53] and interviews with several experts in the ESG research field, an initial set of feature variables was summarized based on the principles of scientific validity and data availability [54].

Step 2: Data collection based on the initial set of feature variables was performed. Following the approach in literature [54], missing values in the initial dataset were handled using interpolation methods, and normalization methods were applied to unify data dimensions.

Step 3: Referring to literature [55], correlation coefficient analysis was used to remove variables with high correlation and weak representativeness, ensuring that the retained indicators possess independence and representativeness.

Step 4: Variables were further refined by eliminating those deemed clearly unimportant according to the F-Score [56] of all features.

Through these steps, a final set of 19 feature variables was obtained, as shown in Table 1. Table 1 categorizes the 19 features into five categories. Company characteristics and governance structure reflect the basic situation and governance level of enterprises. Factors such as company size, shareholder structure, and executive compensation level can influence corporate ESG behaviors and information disclosure, thereby affecting the likelihood of ESG greenwashing. Financial status and performance are core aspects of enterprise operations. A company’s financial health and profitability directly impact its investments and performance in ESG areas. Financial constraints and distress may prompt enterprises to engage in ESG greenwashing to enhance their image and attract investment. Operational efficiency and management levels determine resource utilization efficiency and innovation capacity. Indicators such as management expense ratio, digital transformation, and corporate innovation reflect a company’s operational and management efficiency and capability, thereby influencing its ESG behaviors. Media attention and environmental regulations reflect the stringency of the external regulatory environment, which can pressure companies to engage in greenwashing in their ESG disclosures. Environmental investment, common prosperity, and business credit are areas of focus for the Chinese government and stakeholders. These indicators reflect the efforts and performance of enterprises in social responsibility and sustainable development, which can also exert social responsibility pressure on enterprises, potentially leading to ESG greenwashing behaviors.

**Table 1:** Feature variables for corporate ESG greenwashing prediction.

Category	Feature	Description
**Company characteristics and governance structure**	Firm Size (FS)	The total assets of the enterprise. Larger firm size generally indicates more resources and stronger market competitiveness.
	Shareholding Ratio of the Largest Shareholder (SRLS)	Reflects the concentration of corporate control. A high proportion of the largest shareholder’s holdings often means the company’s decisions are strongly influenced by a few shareholders.
	Shareholding Ratio of the Second Largest Shareholder/ Largest Shareholder (SRSL/LS)	Measures the balance of the shareholding structure. A higher ratio may indicate a more dispersed shareholding structure, reducing the influence of a single shareholder.
	Institutional Investors Shareholding Ratio (IISR)	The proportion of institutional investors in the company’s shareholding structure. A higher proportion usually means stronger market recognition and stability.
	Total Compensation of Top Three Executives (TCTTE)	Reflects the level of executive compensation and incentive mechanisms. Higher total compensation may indicate stronger incentives but can also increase the company’s cost burden.
**Financial health and performance**	Return on Equity (ROE)	Measures the efficiency of a company in generating sales revenue from its assets. Higher turnover rates usually indicate higher operational efficiency.
	Total Asset Growth Rate (TAGR)	An important indicator reflecting the speed of asset expansion of the enterprise.
	Annual Stock Return (ASR)	A key indicator of the company’s profitability, reflecting its ability to generate net profit using shareholders’ equity.
	Financing Constraints (FC)	Represented by the SA index, indicating the constraints and obstacles faced by the enterprise in obtaining external financing, which may stem from market conditions, the company’s credit status, etc.
	Z-Score (ZS)	A comprehensive score measuring the financial health of the company, predicting the likelihood of future financial distress through multiple financial ratios.
	Total Asset Turnover Ratio (TATR)	Reflects the performance of the company’s stock in the market. Higher return rates usually indicate market confidence in the company’s future development.
**Operational and management efficiency**	Management Expense Ratio (MER)	The proportion of management costs to total costs. Lower management expense ratios typically indicate higher management efficiency.
	Digital Transformation (DT)	The degree and effectiveness of the company’s application of digital technology. Successful digital transformation typically enhances operational efficiency and market competitiveness.
	Firm Innovation (FI)	The company’s investment and outcomes in R&D, technology, and product innovation. Companies with strong innovation capabilities generally have stronger market competitiveness and sustainable development capabilities.
**External influence and compliance**	Media Attention (MA)	The level of media attention and its impact on the company. High media attention can increase public influence but also pose higher public opinion risks.
	Environmental Regulation (ER)	The regulations and requirements imposed by the government or regulatory agencies on the company’s environmental behavior. Strict environmental regulations can increase compliance costs but also promote green development.
**Social responsibility and sustainable development**	Environmental Protection Investment (EPI)	The company’s investment in environmental protection and sustainable development. This indicator reflects the company’s sense of social responsibility and long-term development vision.
	Common Prosperity Index (CPI)	The company’s initiatives and achievements in achieving common prosperity, including promoting balanced social and economic development through public welfare activities and social responsibility projects.
	Business Credit (BC)	The company’s credit status in the market. Good business credit helps the company obtain lower-cost financing and better business cooperation opportunities.

### 2.3. Dataset description

**Fig 2:**
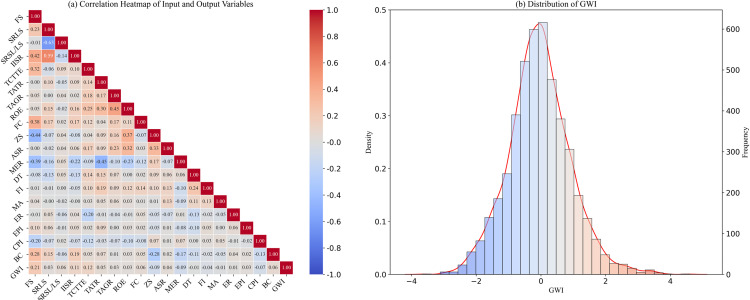
Correlation heatmap of feature variables and distribution of the target variable.

After data collection, calculation of the greenwashing index, and selection of feature variables, the final corporate greenwashing dataset for this study was obtained. To enhance understanding of the data, the dataset is further described as follows:

(1) The dataset comprises samples of Chinese A-share listed companies from 2017 to 2022, which have both Bloomberg ESG ratings and Wind ESG ratings. Companies with unique capital structures in the financial and insurance industries, as well as companies with abnormal financial conditions (ST and * ST companies), are excluded from the sample.
(2) The feature variables in the dataset are listed in Table 1, and the target variable is the GWI. In constructing the dataset, the GWI lags one year behind the feature variables to enable the prediction of the greenwashing index for year *t + *1 using data from year *t*. Therefore, the data years for the greenwashing index are from 2018 to 2022, while the data years for the feature variables are from 2017 to 2021.
(3) The dataset consists of 4584 observations. The data for the feature variables primarily come from Wind and CSMAR databases, while the target variable is calculated using Equation (1). The *ESG*_*dis*_ used in the equation are derived from the Bloomberg ESG Disclosure Scores, and the *ESG*_*per*_ come from the Wind database.
(4) The visual description of the dataset is shown in Fig 2. Fig 2a clearly illustrates the correlations between various variables. Fig 2b displays the distribution of GWI. The results indicate that the GWI roughly follows a normal distribution, with most companies’ GWI values centered around 0, suggesting that most companies do not exhibit significant greenwashing behaviors. However, some companies have GWI values significantly greater than 0, indicating potential significant greenwashing behaviors.

## 3. IHPO-XGBoost model for predicting corporate greenwashing

### 3.1. XGBoost

The basic idea behind XGBoost is to iteratively add new weak learners to fit the residuals of the previous training. The final prediction for each sample is obtained by summing the prediction scores from all weak learners [35]. The main principle of building a corporate greenwashing prediction model based on XGBoost is as follows:

Assume the corporate greenwashing prediction dataset is $D=\left\{(x_{i},y_{i}):i=1,\cdots ,n;x_{i}\in R^{m};y_{i}=R\right\}$,where each sample enterprise *x*_*i*_ has *m* features and corresponds to a target value *y*_*i*_, The greenwashing prediction value for the *i*-th sample, ${\hat{y}}_{i}$, can be expressed as follows:


$${\hat{y}}_{i}=\sum_{k=1}^{K}f_{k}(x_{i}),\;{\text{f}}_{k}\in F$$ (2)

where $f_{k}$ is a regression tree, *F* is the space of all possible regression trees, *K* is the total number of regression trees, and $f_{k}(x_{i})$ is the score calculated by the *k*-th tree for the *i*-th sample enterprise *x*_*i*_.

The objective function of the corporate greenwashing prediction model is defined as:


$$Obj=\sum_{i=1}^{n}l(y_{i},{\hat{y}}_{\text{i}})+\sum_{k=1}^{K}\Omega (f_{k})$$ (3)

where $l(y_{i},{\hat{y}}_{i})$ is the loss function used to measure the fitting degree between the predicted greenwashing values and the actual values, and $\Omega (f_{k})$ is a regularization term used to penalize complex models to avoid overfitting.

By integrating and reorganizing the Taylor expansion of the objective function and converting it into a polynomial related to the prediction residuals, the optimal weight ${\text{w}}_{j}^{*}$ of the leaf nodes and the optimal solution of the objective function value are obtained as follows:


$${\text{w}}_{j}^{*}=-\frac{G_{j}}{H_{j}+\lambda }$$ (4)

$$Obj^{*}=-\frac{1}{2}\sum_{j=1}^{T}\frac{G_{j}^{2}}{H_{j}+\lambda }+\gamma \times T$$ (5)

where $G_{j}=\sum_{i\in I_{j}}g_{i}$, $H_{j}=\sum_{i\in I_{j}}h_{i}$, $g_{i}$ and $h_{i}$ are the first and second derivatives of the loss function, $I_{j}=\{i |\;q(x_{i})=j\}$ is the sample group of leaf node *j*, $q(x_{i})$ is the tree structure function, and $Obj^{*}$ is the tree structure score. A smaller $Obj^{*}$ indicates a lower overall loss. *T* is the number of leaf nodes, *γ* is the penalty coefficient, *λ* represents the L2 regularization term.

To solve for the objective value, a greedy algorithm is used to split the subtrees. The greedy algorithm achieves global optimization by controlling local optimizations. It attempts to add one split point to the existing leaf nodes, enumerates feasible split points, and selects the split with the highest gain [44]. The gain formula is expressed as:


$$\{\begin{array}{l} G=\frac{1}{2}\left[\frac{G_{L}^{2}}{H_{L}+\lambda }+\frac{G_{R}^{2}}{H_{R}+\lambda }+\frac{{\left(G_{L}+G_{R}\right)}^{2}}{H_{L}+H_{R}+\lambda }\right]-\gamma \\ G_{L}=\sum_{i\in I_{L}}{\text{g}}_{i},\;G_{R}=\sum_{i\in I_{R}}{\text{g}}_{i},\;H_{L}=\sum_{i\in I_{L}}{\text{h}}_{i} \end{array}$$ (6)

where $I_{L}$ and $I_{R}$ are the sample sets of the left and right subtrees after the split, $G_{L}^{2}/\left(H_{L}+\lambda \right)$ and $G_{R}^{2}/\left(H_{R}+\lambda \right)$ are the information scores of the left and right subtrees, and ${\left(G_{L}+G_{R}\right)}^{2}/\left(H_{L}+H_{R}+\lambda \right)$ is the information score of the current node before splitting.

### 3.2. IHPO-XGBoost prediction model

XGBoost has numerous hyperparameters, and inaccurate hyperparameter settings can affect the model’s predictive efficiency and effectiveness. However, the hyperparameter optimization process is essentially a black-box function optimization problem. If too many parameters are optimized, the model can become redundant, leading to increased computational complexity and affecting overall system performance [57]. Therefore, this study selects three key parameters for optimization that significantly influence the predictive performance of the XGBoost algorithm: n_estimators, learning_rate, and max_depth [58]. n_estimators specifies the number of weak estimators in the ensemble algorithm. A higher value increases the model’s learning capability but also makes the model more prone to overfitting; max_depth controls the maximum depth of the trees in the model. A higher value increases the model’s complexity and the likelihood of overfitting; learning_rate controls the iteration rate and can prevent overfitting by adjusting the step size during the learning process [59].Yu et al. [35] pointed out that using intelligent optimization algorithms for hyperparameter adjustment not only can obtain the optimal parameter combination but also reduce time and enhance efficiency. Therefore, this study introduces the HPO algorithm, a novel intelligent optimization algorithm, to address the hyperparameter optimization problem for XGBoost.

#### 3.2.1. Original HPO algorithm.

The basic principle of the HPO algorithm proposed by Naruei et al. [46] is as follows:

First, initialize the prey population positions. The initial position *x*_*i*_ of the *i*-thprey in the population is a random number within the range of the lower and upper limits [*l*,*u*], as shown in Equation (7), where *d*is the dimension of the variable. After initializing the population positions, the fitness is determined based on the objective function *f*(*x*).


$$x_{i}=rand(1,d)\times (u_{b}-l_{b})+l_{b}$$ (7)

The hunter search mechanism is described by Equation (8), where *x*_*i,j*_(*t*) represents the hunter’s current position, *x*_*i,j*_(*t + *1) represents the hunter’s next position, and *P*_*pos*_is the prey’s position. The balance parameter *C*is calculated as shown in Equation (9), where *Iter* is the current iteration number of the algorithm, and *MaxIter* is the maximum number of iterations of the algorithm. The adaptive parameter *Z*is calculated as shown in Equation (10), where $\vec{R_{1}}$ and $\vec{R_{3}}$ are random vectors within [0,1], *R*_*2*_ is a random number within [0,1], *P* is the index value of $\vec{R_{1}}<C$, and *IDX* is the index value of $\vec{R_{1}}$ that satisfies the condition of (P==0).


$$x_{i,j}(t+1)=x_{i,j}(t)+0.5\times \left[\left(2\times C\times Z\times P_{pos}-x_{i,j}(t)\right)+\left(2\times (1-C)\times Z\times \bar{x_{i,j}}-x_{i,j}(t)\right)\right]$$ (8)

$$C=1-Iter\times \left(\frac{0.98}{MaxIter}\right)$$ (9)

$$P=\vec{R_{1}}<C;\;IDX=(P==0);\;Z=R_{2}⊗IDX+\vec{R_{3}}⊗(\sim IDX)$$ (10)

The hunter selects the prey farthest from the population’s average position as the target. The Euclidean distance *D*_*euc*_(*i*), for each member is calculated as shown in Equation (11). Considering that after capturing the prey, the hunter continues to move to the new prey position, a decay mechanism is introduced as shown in Equation (13), where *N* is the number of search agents. As the iteration progresses, the prey position $\vec{P_{pos}}$ is continuously updated as shown in Equation (12).


$$D_{euc}(i)=\sqrt{\sum_{j=1}^{d}{\left(x_{i,j}-\bar{x_{i,j}}\right)}^{2}}$$ (11)

k=round(C×N) (12)

$$\vec{P_{pos}}=\vec{x_{i}} |\;i\;is\;sorted\;D_{euc}(k)$$ (13)

The prey position update formula is shown in Equation (14), where *x*_*i,j*_(*t*) represents the current position of the prey, *x*_*i,j*_(*t + *1) represents the next position of the prey, *R*_*4*_ is a random number within [-1,1], and *T*_*pos*_ is the global optimal position. In the process of finding the global optimal solution, the HPO algorithm selects hunters and prey based on the parameters *R*_*5*_ and the adjustment parameter *β*, where *R*_*5*_ is a random number within [0,1]. When *R*_*5*_ < *β*, the search agent is a hunter and updates its position using Equation (8); otherwise, the search agent is a prey and updates its position using Equation (14).


$$x_{i,j}(t+1)=T_{pos}+C\times Z\times \cos(2\pi \times R_{4})\times \left(T_{pos}-x_{i,j}(t)\right)$$ (14)

#### 3.2.2. Improved HPO algorithm.

**(1) Tent chaos mapping and Lévy distribution:** A high-quality initial population helps improve the optimization performance of the algorithm. However, the HPO algorithm uses random initialization, which makes it difficult to ensure the quality of the initial population. Chaos mapping, with characteristics such as randomness, ergodicity, and regularity, can ensure population diversity [60]. Therefore, this study uses sequences generated by Tent Chaos Mapping to initialize the population to enhance early population diversity and improve convergence speed. The expression for Tent Chaos Mapping is as follows:


$$y_{i+1}= \{\begin{array}{l} \eta y_{i},\;0\leq y_{i}<0.5\\ \eta (1-y_{i}),\;0.5\leq y_{i}\leq 1 \end{array}$$ (15)

where *i* is the corresponding particle number, i=1,2,……,N; η∈(0,2] represents the chaotic parameter, which is proportional to chaos. In this study, η=2.

Additionally, random variables following a Lévy distribution [61] are introduced to address the issues of small periodic points and unstable periodic points, ensuring the three properties of the Tent Chaos Mapping sequence. Therefore, the initial values based on Tent Chaos Mapping and the Lévy distribution can be calculated as follows:


$$y_{i+1}= \{\begin{array}{l} \eta y_{i}+\mu \frac{Levy(0,1)}{N},\;0\leq y_{i}\leq 0.5\\ \eta (1-y_{i})+\mu \frac{Levy(0,1)}{N},\;0.5<y_{i}\leq 1\; \end{array}$$ (16)

Substituting Equation (16) into Equation (7) yields the initial population, which can be expressed as:


$$x_{i}=y_{i}\times (u_{b}-l_{b})+l_{b}$$ (17)

**(2) Adaptive inertia weights:** Inertia weight is an important control parameter in intelligent algorithms, including both linear and nonlinear adjustment techniques, to improve the convergence of the algorithm [62]. Nonlinear relationships are widely present in practical optimization problems, making nonlinear strategies more broadly applicable. This allows the algorithm to have good global and local balancing capabilities during the prey iteration phase, accelerating convergence to the optimal solution. In this study, a nonlinear decreasing inertia weight based on a concave function is applied to the HPO algorithm, which can be expressed as follows:


$$w=w_{\min}{\left(\frac{w_{\max}}{w_{\min}}\right)}^{\frac{1}{\frac{1+c\times Iter}{MaxIter}}}$$ (18)

Where $w_{\min}$ and $w_{\max}$ are the maximum and minimum inertia weights, respectively, and *c* is the adjustment parameter.

Incorporating Equation (18) into Equation (14) yields Equation (19) as the new prey position update strategy:


$$x_{i,j}(t+1)=w\times T_{pos}+C\times Z\times \cos(2\pi \times R_{4})\times \left(T_{pos}-x_{i,j}(t)\right)$$ (19)

#### 3.2.3. Construction of the IHPO-XGBoost model.

**Fig 3:**
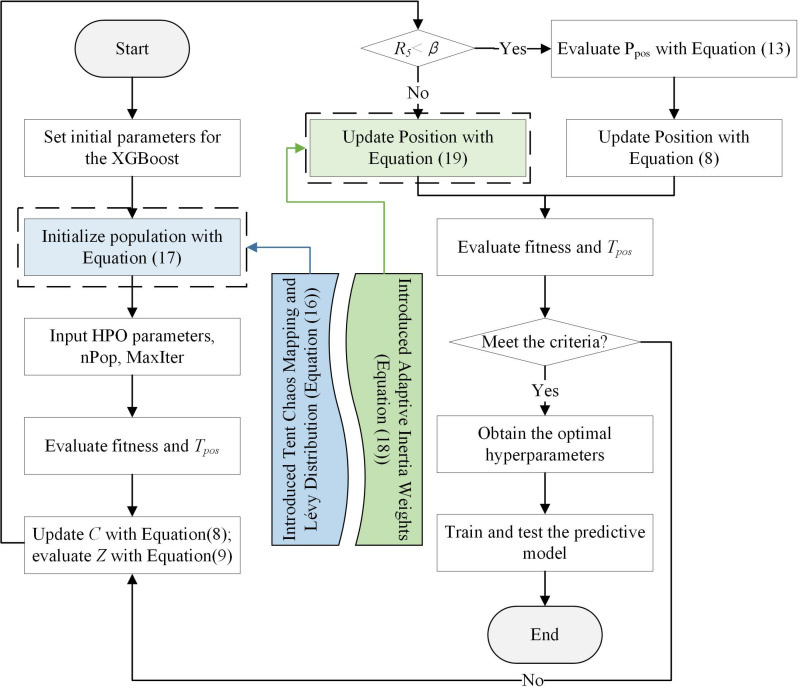
Flowchart of IHPO-XGBoost.

Utilizing the optimization capabilities of the IHPO algorithm and the learning capabilities of the XGBoost algorithm, this study proposes an IHPO-XGBoost prediction model optimized by the IHPO algorithm. The flowchart of the model is shown in Fig 3.

This model aims to improve the predictive performance of the XGBoost algorithm by finding the optimal set of parameters during the training process, minimizing the error between the predicted results and actual values. According to Fig 3, the implementation process of the IHPO-XGBoost is as follows:

Step 1: Set the initial parameters of the XGBoost algorithm, including the parameter ranges and initial values, such as n_estimators, learning_rate, and max_depth.

Step 2: Based on Equation (17), use the Tent Chaos Mapping method combined with the Lévy Distribution to initialize the population, ensuring the diversity and high quality of the initial population to enhance the exploration capability of the algorithm.

Step 3: Set the key parameters of the HPO algorithm, including the population size (nPop) and the *MaxIter*.

Step 4: Evaluate the fitness and *T*_*pos*_. Use the defined fitness function to evaluate the fitness of the initial population. This function is based on the performance of the XGBoost model, using cross-validation to ensure the robustness of the fitness evaluation.

Step 5: Update *C* using Equation (9); evaluate *Z* using Equation (10).

Step 6: Select the appropriate position update strategy based on whether *R*_*5*_<*β*.

Step 7: Reevaluate the fitness of the population and *T*_*pos*_ after the position updates, and determine if the algorithm meets the convergence criteria. If the criteria are met, the optimization process terminates; otherwise, continue to the next iteration.

Step 8: Use the optimal hyperparameters (n_estimators, learning_rate, and max_depth) obtained by the IHPO algorithm to train and test the corporate greenwashing prediction model using XGBoost.

## 4. Performance evaluation and interpretability of model

### 4.1. Model performance evaluation

Following the approach in [55,63,64], this study evaluates the model’s performance using the coefficient of determination (*R*^*2*^), Root Mean Squared Error (*RMSE*), Mean Absolute Error (*MAE*), and *Adjusted R*^*2*^. *R²* represents the percentage of the variance in the dependent variable that is predictable from the independent variables. It characterizes the degree of regression fit to all research samples and is commonly used as the primary indicator of prediction accuracy in regression problems [65]. *RMSE* and *MAE* are crucial indicators for measuring model error, while *Adjusted R*^*2*^penalizes the number of input features to adjust for potential overfitting in *R²* [66]. The specific calculation formulas for these four indicators are as follows:


$$R^{2}=1-\frac{\sum_{i=1}^{n}{\left(y_{i}-{\hat{y}}_{i}\right)}^{2}}{\sum_{i=1}^{n}{\left(y_{i}-\bar{y}\right)}^{2}}$$ (19)

$$RMSE=\sqrt{\frac{1}{n}\sum_{i=1}^{n}{\left(y_{i}-{\hat{y}}_{i}\right)}^{2}}$$ (20)

$$MAE=\frac{1}{n}\sum_{i=1}^{n}\left|y_{i}-{\hat{y}}_{i}\right|$$ (21)

$$A\text{d}justed\;R^{2}=1-\left(\frac{\left(1-R^{2})\times (n-1\right)}{\left(n-p-1\right)}\right)$$ (22)

Where $y_{i}$ represents the actual value, ${\hat{y}}_{i}$ represents the predicted value, $\bar{y}$ represents the mean value, *n* represents the number of samples, and *p* represents the number of features. The higher the *R*^*2*^ and *Adjusted R*^*2*^, and the lower the *RMSE* and *MAE*, the better the model performance and the smaller the gap between the predicted and actual values.

### 4.2. Model interpretability

SHAP is an algorithm for interpreting machine learning models, primarily based on cooperative game theory to compute Shapley values. These values assess the contribution of each feature to the model’s prediction, thereby explaining the output of the machine learning model [35]. The principle of SHAP is as follows.

Assume the *i* -th enterprise sample is $x_{i}$, and the SHAP value $\phi_{x_{i,j}}$ of its *j* -th feature $x_{i,j}$ is calculated as follows:


$$\phi_{x_{i,j}}=\sum_{S\in M\backslash \{x_{i,j}\}}\frac{\left|S\right|!(m-\left|S\right|-1)!}{m!}\left[f_{x_{i}}(S\cup \{x_{i,j}\})-f_{x_{i}}(S)\right]$$ (23)

where *M* is the set of all features in the enterprise greenwashing behavior dataset with a dimension of *m*; *S* is a subset drawn from *M* with a size of $\left|S\right|$; $f_{x_{i}}(S)$ is the model’s prediction for the enterprise sample $x_{i}$ using only the feature set, and when *S* is an empty set, the value of $f_{x_{i}}(S)$ is considered the baseline, i.e., the average prediction of the model across all enterprise samples; $f_{x_{i}}(S\cup \{x_{i,j}\})$ is the model’s average prediction for the sample $x_{i}$ across all samples when the feature value $x_{i,j}$ is added to the feature set *S*; $M\backslash \{x_{i,j}\}$ represents the subset of all features excluding the *j* -th feature.

Based on SHAP values, the SHAP explanation for the enterprise greenwashing behavior prediction model can be expressed with an additive model as follows:


$$f(x_{i})=\phi_{x_{i,0}}+\sum_{j=1}^{m}\phi_{x_{i,j}}z_{j}$$ (24)

where $\phi_{x_{i,0}}$ is the baseline output of the entire enterprise greenwashing behavior prediction model, i.e., the average prediction of all training samples; $\phi_{x_{i,j}}$ is the SHAP value, representing the contribution of the *j* -th feature of the *i* -thsample to the model output $f(x_{i})$; $z_{j}$ is the simplified feature indicator, which takes the value of 0 or 1, where $z_{j}=1$ indicates that the feature is present in the sample being explained $x_{i}$; $z_{j}=0$ indicates that the feature is not present in $x_{i}$.

For the SHAP value $\phi_{x_{i,j}}$ in the above equation, when $\phi_{x_{i,j}}>0$, it means that the *j* -th feature of the *i* -th enterprise sample increases the model’s prediction value, having a positive effect on the model output; when $\phi_{x_{i,j}}<0$, it indicates that the feature decreases the prediction value, having a negative effect on the model output. Hence, the role of the SHAP algorithm is to collectively use the contribution values of each feature to drive the model’s prediction result from the baseline value to the final model prediction value.

## 5. Results and discussion

### 5.1. Model prediction results

In this study, the constructed corporate greenwashing dataset is split into 80% training set and 20% test set. A five-fold cross-validation method is used for model training, which helps ensure that the model generalizes well to unseen data. To further prevent overfitting, the XGBoost model incorporates regularization parameters, specifically alpha (L1 regularization) and lambda (L2 regularization). The default values for these regularization parameters are used in this study, which helps prevent excessive fitting while ensuring the model’s generalization ability. The operating environment of the model is shown in Table 2. The hyperparameters of the XGBoost algorithm, namely n_estimators, learning_rate, and max_depth, are optimized using the IHPO algorithm. Based on a review of existing research on XGBoost hyperparameter optimization [41–45], it is found that the optimized ranges for these three parameters typically fall within [100, 1000], [0.01, 1], and [1,10], respectively. Therefore, these ranges were set as the initial bounds for optimization. Furthermore, in the IHPO algorithm, through multiple rounds of code tuning, we determined that a population size of 20 and 100 iterations produced the best results. Mean Squared Error (MSE) is used as the fitness function. The final optimized results are n_estimators of 450, learning_rate of 0.1, and max_depth of 6.

**Table 2:** Operating environment of the corporate greenwashing prediction project.

Category	Subcategory	Details
**Hardware**	Processor	AMD Ryzen 7 6800H with Radeon Graphics @ 3.20 GHz
	Memory	16.0 GB DDR4
**Operating System**	Operating System	Windows 11 Home Edition
**Software**	Software	Python 3.8.19
	Development Environment	Jupyter Notebook, Visual Studio Code
	Machine Learning Library	XGBoost 1.4.2, Scikit-Learn 0.24.1
	Data Processing Library	Pandas 1.1.5, NumPy 1.19.5
	Visualization Tools	Matplotlib 3.3.4, Seaborn 0.11.1, SHAP 0.41.0

**Fig 4:**
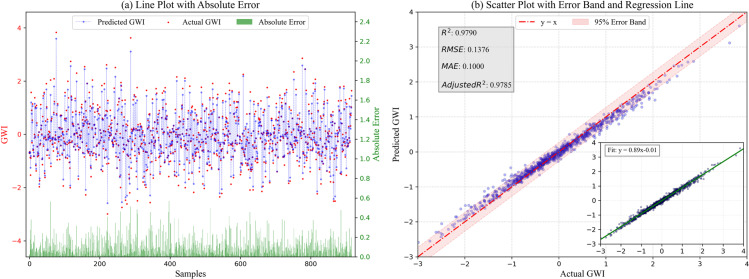
Performance of IHPO-XGBoost model on the test set.

After completing model training, the test set is used to validate the model’s predictive performance, and the prediction results on the test set are visualized (as shown in Fig 4). From Fig 4a, it can be seen that the predicted values of the sample points are very close to the actual values, and the absolute errors are relatively stable, mainly concentrated in the low error range of 0 to 0.2, indicating that the model’s prediction performance is good. Fig 4b shows the scatter plot of the actual values versus the predicted values of the greenwashing index. The vast majority of sample points are distributed within the error band of the 95% confidence interval drawn based on the ideal fit line *y = x*, and the actual fitting equation for all scatter points is y=0.89x−0.01, showing a strong correlation between the predicted and actual values. Additionally, Fig 4b presents the performance evaluation metrics of the IHPO-XGBoost model: *R²*, *RMSE*, *MAE*, and *Adjusted R²* are 0.9790, 0.1376, 0.1000, and 0.9785, respectively. The results presented in Fig 4 demonstrate the excellent predictive performance of the IHPO-XGBoost model.

### 5.2. Comparative analysis of model performance

**Fig 5:**
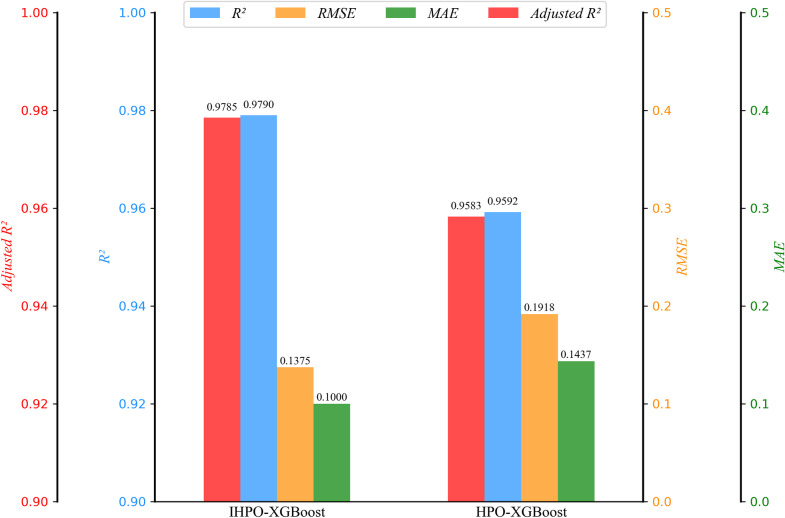
Comparison of model performance before and after HPO improvement.

**Fig 6:**
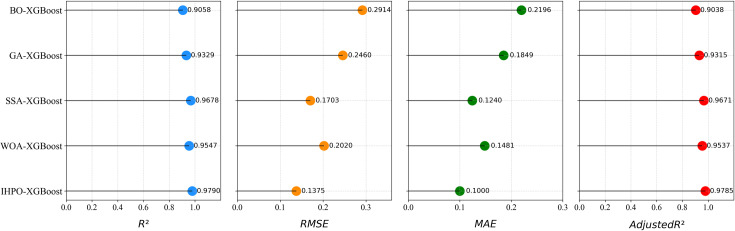
Model performance comparison based on *R²*, *RMSE*, *MAE* and *Adjusted R².*

To verify the effectiveness of the improvements made to the HPO algorithm, the performance of IHPO-XGBoost is compared with HPO-XGBoost on the test set, as shown in Fig 5. The results indicate that using the improved HPO algorithm for hyperparameter optimization of XGBoost leads to certain improvements in *R²* and *Adjusted R²* metrics while significantly reducing *RMSE* and *MAE*. This demonstrates the effectiveness of the proposed optimization strategy.To further validate the superiority of the IHPO-XGBoost model, this study compares the proposed model with WOA-XGBoost [67], SSA-XGBoost [68], GA-XGBoost [69], and BO-XGBoost [70] models on the test set. As shown in Fig 6, the IHPO-XGBoost model exhibits the highest *R²* and 与*Adjusted R²* values, along with the lowest *RMSE* and *MAE*, indicating that the IHPO-XGBoost model has the best overall performance.

### 5.3. Interpretability analysis based on SHAP theory

#### 5.3.1. Global impact analysis of sample features.

**Fig 7:**
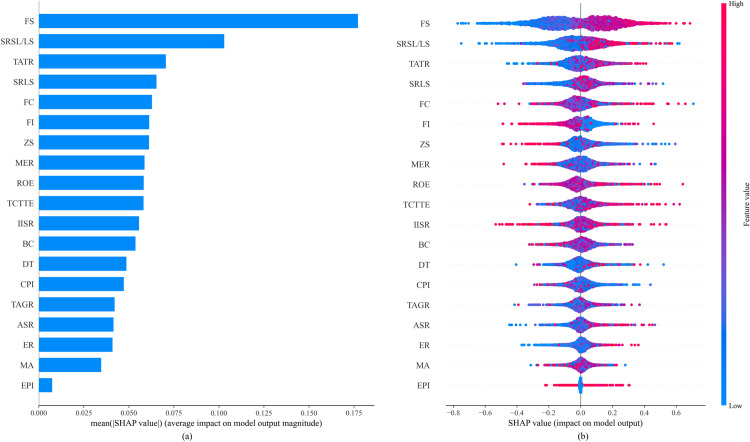
Global model explanation results by SHAP. **(a)** Feature importance analysis. **(b)** SHAP value distribution. Note: Firm Size (FS), Shareholding Ratio of the Second Largest Shareholder/ Largest Shareholder (SRSL/LS), Total Asset Turnover Ratio (TATR), Shareholding Ratio of the Largest Shareholder (SRLS), Financing Constraints (FC), Firm Innovation (FI), Z-Score (ZS), Management Expense Ratio (MER), Return on Equity (ROE), Total Compensation of Top Three Executives (TCTTE), Institutional Investors Shareholding Ratio (IISR), Business Credit (BC), Digital Transformation (DT), Common Prosperity Index (CPI), Total Asset Growth Rate (TAGR), Annual Stock Return (ASR), Environmental Regulation (ER), Media Attention (MA), Environmental Protection Investment (EPI).

Fig 7 presents the visualization of the sample feature global explanation of the IHPO-XGBoost model’s prediction results based on SHAP theory.

Firstly, Fig 7a visualizes the importance of features by showing the mean absolute SHAP values for each feature; the larger the value, the more important the feature. As shown in Fig 7a, overall, features such as FS, SRSL/LS, and TATR have a significant impact on the prediction results, whereas features like EPI are relatively less important. Among them, FS has the greatest impact on the prediction results, with an average absolute SHAP value of 0.1772, indicating that it plays a crucial role in predicting corporate greenwashing. It is followed by SRSL/LS, with an average absolute SHAP value of 0.1030, which also has a significant impact on the prediction results. Other features with significant impacts include TATR, SRLS, and FC, with average absolute SHAP values of 0.0706, 0.0654, and 0.0628, respectively. In contrast, EPI has an average absolute SHAP value of only 0.0074, indicating its minimal impact in the model. This implies that financial status is more important than environmental protection investment in predicting corporate greenwashing.

Fig 7b is the SHAP summary plot for features, showing the distribution of SHAP values for each feature and their corresponding impact trends. In the Fig, the X-axis represents specific SHAP values, and the Y-axis represents input features sorted by importance. The dots in the plot represent samples from the database, with dot colors indicating specific feature values, ranging from blue (low values) to red (high values). The horizontal position of the dots indicates whether the feature values lead to an increase or decrease in the prediction value. For example, the red dots at the top of the Fig indicate that higher FS values lead to a significant increase in the prediction value. Therefore, the SHAP summary plot not only provides an understanding of which features are important but also shows how each feature affects the prediction results. Generally, as the values of features such as FS, SRSL/LS, and TATR increase, the prediction value for corporate greenwashing also increases. In contrast, the increase in features like EPI has a smaller impact on the prediction results.

#### 5.3.2. Interaction impact analysis of features.

**Fig 8:**
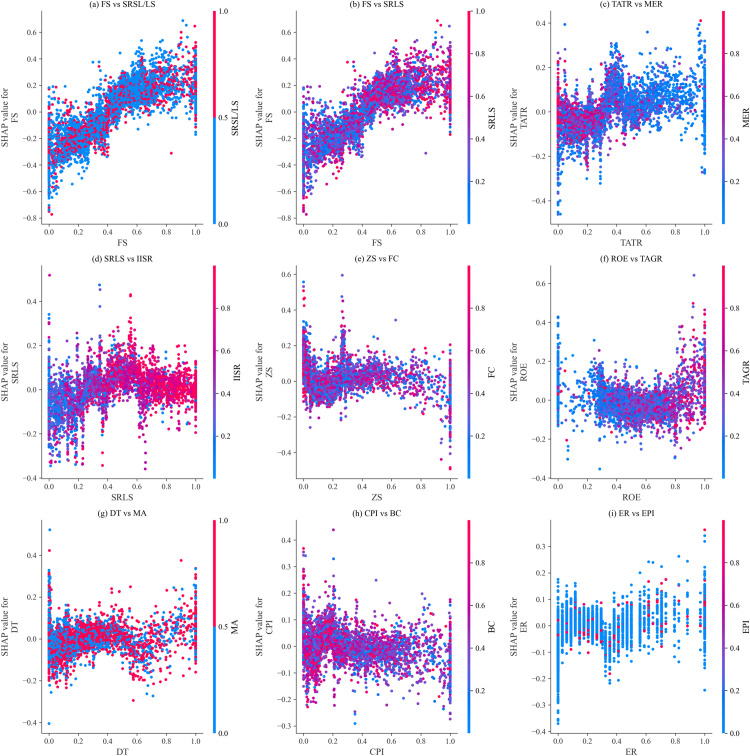
Interaction impact analysis of features on model prediction results. Firm Size (FS), Shareholding Ratio of the Second Largest Shareholder/ Largest Shareholder (SRSL/LS), Total Asset Turnover Ratio (TATR), Shareholding Ratio of the Largest Shareholder (SRLS), Financing Constraints (FC), Firm Innovation (FI), Z-Score (ZS), Management Expense Ratio (MER), Return on Equity (ROE), Total Compensation of Top Three Executives (TCTTE), Institutional Investors Shareholding Ratio (IISR), Business Credit (BC), Digital Transformation (DT), Common Prosperity Index (CPI), Total Asset Growth Rate (TAGR), Annual Stock Return (ASR), Environmental Regulation (ER), Media Attention (MA), Environmental Protection Investment (EPI).

In this section, based on the model importance shown in Fig 7 and actual business significance, nine key feature pairs are selected to analyze their interaction effects, revealing how these features jointly affect the prediction results of corporate ESG greenwashing behavior. The interaction impact of two input features on the model’s prediction results can be visualized using the SHAP dependence plot shown in Fig 8. In Fig 8, the horizontal axis represents the feature values of a certain input feature, the left vertical axis represents the SHAP values corresponding to that input feature, and the right vertical axis represents the feature values of another input feature marked by color, ranging from blue to red indicating low to high feature values.

Based on Fig 8, the analysis and discussion are as follows:

(1) FS, TATR, SRLS, and ER all show a trend where increasing feature values correspond to increasing SHAP values. This indicates that higher values of these features have a more positive impact on the prediction results for corporate ESG greenwashing. Specifically, larger companies (higher FS) have more resources and capabilities, making them more likely to engage in ESG greenwashing. Similarly, companies with high asset utilization efficiency (higher TATR) and high governance concentration (higher SRLS) are more inclined to showcase their management and decision-making advantages through ESG greenwashing. Strict environmental regulations (higher ER) prompt companies to actively engage in ESG greenwashing to meet regulatory requirements and reduce regulatory pressure.
(2) ZS and CPI show a trend where increasing feature values correspond to decreasing SHAP values, indicating that lower values of these features have a more positive impact on the prediction results for corporate ESG greenwashing. Companies with poorer financial health (lower ZS) may use ESG greenwashing to cover up internal issues and enhance external trust. Similarly, companies with lower CPI, indicating poorer social responsibility performance, may use ESG greenwashing to improve their image.
(3) ROE and DT exhibit fluctuating trends. When ROE increases from 0 to 0.8, SHAP values decrease, indicating that in the early stages of profitability, companies focus more on genuine performance improvements. When ROE increases from 0.8 to 1, SHAP values rise, suggesting that in the high profitability stage, companies may increase ESG greenwashing to enhance shareholder satisfaction. For DT, SHAP values increase as the feature value rises from 0 to 0.4, indicating a positive impact of initial digital transformation on ESG greenwashing. SHAP values then decrease from 0.4 to 0.6, possibly due to challenges in technology integration and organizational change. When DT exceeds 0.8, SHAP values rise again, indicating that deep digital transformation enables companies to better utilize digital means for ESG greenwashing.
(4) Additionally, Fig 8 reveals clear relationships between certain features. For example, higher FS may correspond to higher SRLS, which is further related to higher IISR; high ROE is usually accompanied by high TAGR. Conversely, lower TATR often correlates with higher MER, while lower ZS is associated with higher FC, and low CPI may correspond to high BC. These relationships reveal complex interactions between features, helping us to understand corporate ESG greenwashing behavior in different contexts more deeply. For instance, higher FS typically implies higher SRLS, which may reflect a more concentrated governance structure and higher decision-making efficiency, thus leading to a greater tendency for ESG greenwashing. Similarly, the association between high ROE and high TAGR suggests that highly profitable companies may be more motivated to engage in ESG greenwashing to enhance their financial performance when expanding assets. The relationship between lower TATR and higher MER may indicate that despite lower asset turnover, companies with higher MER may still showcase their management capabilities through ESG greenwashing. Finally, the association between lower ZS and higher FC implies that companies with poorer financial health are more likely to face financing constraints and therefore may engage in ESG greenwashing to improve external perceptions of their financial status.

#### 5.3.3. Single sample feature impact analysis.

**Fig 9:**
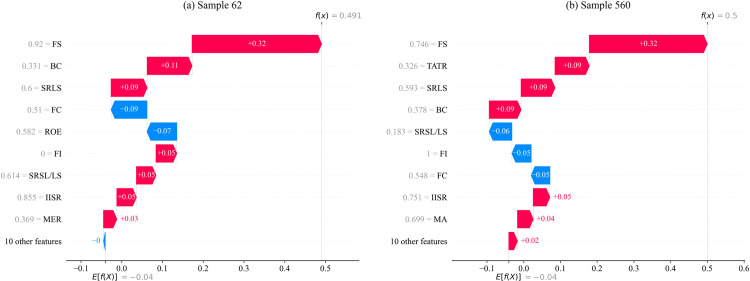
SHAP explanation of predictions for single samples. Firm Size (FS), Shareholding Ratio of the Second Largest Shareholder/ Largest Shareholder (SRSL/LS), Total Asset Turnover Ratio (TATR), Shareholding Ratio of the Largest Shareholder (SRLS), Financing Constraints (FC), Firm Innovation (FI), Management Expense Ratio (MER), Return on Equity (ROE), Institutional Investors Shareholding Ratio (IISR), Business Credit (BC), Media Attention (MA).

Two samples with predicted values around 0.5 are selected from the test set to analyze the impact of input features on the prediction results using SHAP theory, as shown in Fig 9. In the Fig, the horizontal axis represents the size of the predicted value, and the vertical axis represents the value of the input feature. Red indicates that the SHAP value of the input feature is positive, while blue indicates that the SHAP value is negative. The larger the area of the red and blue regions, the greater the SHAP value, and thus the greater the impact on the prediction result for the sample.

In Fig 9a, the predicted value for sample 62 is 0.491. FS has the greatest impact on the prediction result for sample 62, with a SHAP value of + 0.32, indicating that a larger FS positively drives the prediction value. Additionally, BC and SRLS also have positive impacts on the prediction result, with SHAP values of + 0.11 and + 0.09, respectively, showing the positive contributions of market trust and shareholder structure to the company’s ESG performance. On the other hand, FC has a negative impact on the prediction value, with a SHAP value of -0.09, indicating that financial constraints inhibit the prediction value increase for this sample. Overall, the prediction result for sample 62 is primarily driven by the positive influences of FS and BC, but the negative impact of FC cannot be ignored.

In Fig 9b, the predicted value for sample 560 is 0.5. FS also has the greatest impact on the prediction result for this sample, with a SHAP value of + 0.32. TATR and SRLS have significant positive impacts on the prediction result, with SHAP values of + 0.09 and + 0.09, respectively, indicating good performance in asset utilization efficiency and shareholder structure. However, SRSL/LS and FC have negative impacts on the prediction value, with SHAP values of -0.06 and -0.05, respectively, showing that these factors suppress the prediction value for this sample. It can be seen that the prediction result for sample 560 is primarily driven by the positive influences of FS and TATR, but the negative impacts of SRSL/LS and FC also play a role in inhibiting the prediction result.

The SHAP value analysis of the two samples with similar predicted values in Fig 9 reveals that different features have varying impacts on different samples. Therefore, explanations of input features should not be overly absolute to avoid misinterpretations.

## 6. Conclusion

This study investigates the issue of predicting corporate greenwashing behavior using a dataset of A-share listed companies from the Shanghai and Shenzhen stock exchanges between 2017 and 2022. A comprehensive dataset for predicting corporate greenwashing was first constructed. Then, an IHPO-XGBoost ensemble learning model was proposed to predict corporate ESG greenwashing behavior, with its effectiveness validated through comparative analysis. Finally, SHAP theory was employed to explain the model’s prediction results. The main findings of this study are as follows:

**(1) Superior performance of the IHPO-XGBoost model:** The model achieved *R*², *RMSE*, *MAE*, and *adjusted R*² scores of 0.9790, 0.1376, 0.1000, and 0.9785, respectively, demonstrating high accuracy and stability in predicting corporate ESG greenwashing behavior. The IHPO-XGBoost model outperforms the traditional HPO-XGBoost model across multiple metrics, confirming the effectiveness of the IHPO algorithm in hyperparameter optimization. Furthermore, compared to other optimization algorithms (such as WOA, SSA, GA, and BO) in combination with XGBoost, IHPO-XGBoost exhibits the best overall performance.

**(2) Insights from SHAP theory:** The application of SHAP theory enabled a more comprehensive understanding of the model’s predictions. The global explanation of sample features revealed that factors such as Firm Size, Shareholder Structure, and Total Asset Turnover Ratio play crucial roles in predicting corporate ESG greenwashing behavior. The feature interaction analysis demonstrated that the interplay between specific features significantly influences model predictions, helping to uncover the complex decision-making processes behind corporate ESG practices. The single-sample feature impact analysis further clarified the contribution of each feature to the prediction results, offering valuable insights into the context-specific dynamics of corporate ESG performance.

**(3) Practical implications:** The results have important implications for regulatory agencies and investors seeking to identify and assess corporate ESG greenwashing behavior. Understanding the key features and their interactions can facilitate more effective monitoring of corporate ESG disclosures, promoting transparency and reducing the risk of greenwashing. Corporate managers can also leverage these findings to optimize their ESG strategies, enhancing their sustainability efforts.

In conclusion, the IHPO-XGBoost model, combined with SHAP theory, provides a robust framework for predicting and managing corporate ESG greenwashing behavior. Future research could extend this analysis by incorporating longer time spans to explore the evolution of corporate ESG practices and by including additional data sources, such as social media and news reports, to improve the model’s predictive power and comprehensiveness.

## Supporting information

S1 Dataset ESG greenwashing prediction dataset. (XLSX)
